# Elevated Aromatase (*CYP19A1*) Expression Is Associated with a Poor Survival of Patients with Estrogen Receptor Positive Breast Cancer

**DOI:** 10.1007/s12672-017-0317-2

**Published:** 2018-01-23

**Authors:** Andrea Friesenhengst, Tamara Pribitzer-Winner, Heidi Miedl, Katharina Pröstling, Martin Schreiber

**Affiliations:** 1 Department of Obstetrics & Gynecology, Medical University of Vienna, Waehringer Guertel 18-20/5Q, 1090 Vienna, Austria; 2 University of Applied Sciences, 1030 Vienna, Austria; 3 Comprehensive Cancer Center, Medical University of Vienna, 1090 Vienna, Austria

**Keywords:** Breast Cancer, CYP3A5 Expressers, ESR1 mRNA Expression, CYP1A1 mRNA Levels, Mean CYP3A

## Abstract

Genetic variants in *CYP19A1*, the gene encoding aromatase, have been reported to be associated with circulating estrogen concentrations, a key risk factor for breast cancer. The mechanism underlying this association is still unclear; it has been suggested that some of these variants may alter the expression and/or activity of aromatase. Here we analyzed the expression of intra-tumoral *CYP19A1* messenger RNA (mRNA) and the genotypes of rs10046, a well-characterized single nucleotide polymorphism in *CYP19A1*, in 138 breast cancer patients and 15 breast cancer cell lines. The genotype TT was detected in 36 patients and six cell lines, genotype CT in 55 patients and five cell lines, and genotype CC in 28 patients and four cell lines. We found no evidence for a significant association of *CYP19A1* levels with rs10046 genotypes, although expression tended to be higher in tumors and cell lines with the homozygous risk genotype TT. We also found no evidence for a significant association of rs10046 genotypes with breast cancer prognosis. In contrast, high *CYP19A1* expression was highly significantly associated with a poor overall, disease-free, and metastasis-free survival in estrogen receptor-positive but not negative breast cancer patients. Moreover, *CYP19A1* mRNA was significantly elevated in postmenopausal patients and in patients older than 50 years, and a trend towards a positive correlation with ER status and *ESR1* mRNA expression was observed. These findings highlight the key role of aromatase in estrogen receptor-positive breast cancer biology.

**Electronic supplementary material:**

The online version of this article (10.1007/s12672-017-0317-2) contains supplementary material, which is available to authorized users.

## Introduction

*CYP19A1*, a member of the cytochrome P450 gene superfamily, encodes the enzyme aromatase, which catalyzes the last, rate-limiting step in estrogen biosynthesis. By sequential hydroxylation, removal of the axial C-19 methyl group, and aromatization of the A-ring, aromatase converts the androgens testosterone and androstenedione to estradiol and estrone, respectively [1]. Lifetime exposure to elevated concentrations of circulating estrogen is an established risk factor for breast cancer, as are aspects of physiology and lifestyle associated with these elevated levels, such as early menarche, late menopause, nulliparity, high BMI, oral contraceptives, and hormone replacement therapy [2–5]. Estrogen biosynthesis takes place primarily in the ovaries of premenopausal women but switches to extra-gonadal sites such as adipose tissue, skin, and muscle after menopause, concomitant with the sites of highest aromatase expression [1]. Aromatase is frequently overexpressed by breast tumor and adjacent stroma cells [6, 7].

Aromatase inhibitors such as anastrozole, letrozole, and exemestane are highly effective endocrine therapies of postmenopausal, estrogen receptor (ER)-positive breast cancer, prolonging the disease-free survival (DFS) and overall survival (OS) [8–10]. Conversely, high expression of aromatase has been associated with a shortened survival, albeit inconsistently [11–18]. Unfortunately, only few of these studies have specifically analyzed ER-positive patients [16, 19].

Genetic variants of *CYP19A1* have been associated with elevated circulating estrogen levels in pre- and postmenopausal women and also in men [3, 20–23]. Per risk allele, the estradiol/testosterone ratio was found increased by about 10%, which explains ~ 1.1–1.6% of its variance in postmenopausal women [3, 23]. By comparison, the BMI accounts for ~ 16% of the variance of circulating estrogen levels, and the estimated overall heritability is about 40% [23, 24]. Since elevated levels of circulating estrogen increase the risk of breast cancer and other hormone-dependent cancers, it seems plausible that *CYP19A1* variants associated with elevated estrogen levels should also be associated with an increased risk of these cancers. However, whereas such an association has been well-established for endometrial cancer [23], most *CYP19A1* candidate SNP studies did not find an association with breast cancer risk [25–31]. This may be due to differences in effect size: the odds ratio associated with a doubling of circulating estradiol levels has been estimated to be 2.06 (95% CI, 1.47–2.89) for endometrial cancer, but only 1.29 (95% CI, 1.15–1.44) for breast cancer [2, 32].

The potential association of *CYP19A1* variants with breast cancer prognosis has also been investigated in detail. The CC genotype of rs10046, one of the most extensively studied *CYP19A1* candidate SNPs, was found associated with a longer disease-free survival of premenopausal, but not postmenopausal breast cancer patients [33]. Likewise, decreased OS and DFS were found for the minor allele of rs700519, which is in linkage disequilibrium with rs10046, plus five other *CYP19A1* SNPs [34]. Another *CYP19A1* haplotype was associated with a poor OS, DFS, and metastasis-free survival (MFS) in premenopausal, ER-positive breast cancer patients [35]. In contrast, two other large studies found no evidence for an association of rs10046 and OS, DFS, and MFS in unselected breast cancer patients [36, 37].

The aim of our study was to analyze the association of *CYP19A1* messenger RNA (mRNA) expression and the common SNP rs10046 with prognosis specifically in ER-positive vs. ER-negative breast cancer patients. Moreover, we wanted to investigate the association of *CYP19A1* expression with widely used clinical and histopathological characteristics of breast cancer, in particular the ER status and the age at onset. Finally, by determining the association of rs10046 genotypes with *CYP19A1* expression, we addressed a proposed mechanism by which genetic variants in *CYP19A1* might affect circulating estrogen levels and (breast) cancer risk.

## Patients and Methods

### Study Population

This study was approved and is annually reviewed by the Institutional Review Board (IRB, “Ethikkommission”) of the Medical University of Vienna, Austria (MUV; protocol 141/2002). Written informed consent was obtained from patients recruited after the onset of the study. For those patients who had undergone surgery before the onset of the study, a waiver of specific informed consent was approved by the IRB. One hundred and thirty-eight consecutive breast cancer patients treated between 1991 and 1994 at the Department of Obstetrics and Gynecology, MUV, were enrolled in this study. Detailed follow-up records and fresh-frozen tumor tissue were available for each patient. Only women of Western European descent from the same geographical area were included. Clinical and histopathological characteristics of the study population are shown in Supplementary Table 1. Molecular subtypes were defined based on IHC analyses as follows: Triple negative, ER−, PR−, and HER2−; HER2-enriched, ER−, PR−, and HER2+; Luminal A, ER+ and/or PR+, HER2−; and Luminal B, ER+ and/or PR+, HER2+. None of the patients received any neoadjuvant therapy, or any treatment with aromatase inhibitors, or any treatment prior to tumor tissue isolation. Thiry-five patients received adjuvant chemotherapy, 39 received tamoxifen anti-hormonal therapy, 37 received both, and 21 received no systemic therapy. For six patients, the records are incomplete. Of the 72 patients with chemotherapy, the schemes were CMF (*n* = 36), modified CMF (*n* = 9), or other (*n* = 27). Sixty-one patients were ER positive. Forty-seven of those were treated with tamoxifen, and 14 were not. Of the latter, four received chemotherapy, and ten no systemic therapy.

### DNA Isolation and Genotyping

Genomic DNA from 122 patients has been extracted previously from fresh-frozen primary tumor samples with the High Pure PCR Template Preparation Kit (Roche, Vienna, Austria) as described [38]. Genotyping of SNP rs10046 (CYP19 E10 c.+19C>T) was performed by TaqMan PCR with Genotyping Master mix and allele-specific, fluorescently labeled probes on a 7500 fast instrument following the manufacturer’s instructions (Applied Biosystems, Brunn/Gebirge, Austria; Assay-ID # C___8234731_30). Twenty nanograms of genomic DNA was used per reaction in a reaction volume of 10 μl.

### qRT-PCR Analysis

Isolation of total RNA from 111 fresh-frozen tumor samples with TRIreagent (Sigma), quality control with the Bioanalyser 2100 (Agilent), and Reverse Transcription with the High-Capacity complementary (cDNA) Archive Kit (Applied Biosystems, Brunn/Gebirge, Austria) has been described previously [38, 39]. For 17 patients, *CYP19A1* mRNA levels were also determined in one lymph node metastasis each in addition to the primary tumor. In each of our qRT-PCR runs, which were conducted in 96-well plates, two to four negative controls (2.5 μl ddH2O instead of cDNA) were included and run in parallel. No signal was obtained in any of these reactions. As a positive control, duplicate samples of serial dilutions of a cDNA standard (cultured normal breast epithelial cells; HMECs) were included in each run. Each sample was analyzed in duplicate by quantitative reverse transcription PCR (qRT-PCR) on an Applied Biosystems 7500 fast real-time PCR instrument, using the following gene-specific primers and fluorescent probes obtained from Applied Biosystems: *CYP19A1* (aromatase), hs_00903413; *ESR1* (estrogen receptor 1; ERα), hs_00174860; *CDH1* (E-cadherin), hs_00170423; and *ACTB1* (β-actin; control), hs_99999903. The mRNA levels of *CYP19A1*, *ESR1*, and *CDH1* were normalized to those of *ACTB1* in each sample and were further normalized to controls by setting the mean level of four samples of normal breast tissue to unity (1) and expressing the levels of all other samples relative to those. For expression analyses in breast cancer cell lines, four non-cancer cell lines were used as normalization controls (HMEC, Hs578Bst, MCF10A, and MCF10F). Thus determined *CDH1* mRNA levels in breast cancer cell lines were assigned to a positive or negative *CDH1* status by setting the mean level of the non-cancer cell lines HMEC, MCF10A, and MCF10F to unity and defining cell lines with at least one tenth that level as positive and all others as negative. Of note, we found a large difference of at least 75-fold between CDH1-positive and CDH1-negative cell lines (data not shown). All relative mRNA expression levels are presented as − ΔΔCt values (i.e., as log(2) values) as described [38].

### Cell Lines

Finite lifespan untransformed human mammary epithelial cells (HMEC) were kindly provided by M. R. Stampfer and grown in MEGM medium [40]. All other cell lines were purchased from DSMZ (“Deutsche Sammlung von Mikro-Organismen und Zellkulturen”) or ATCC (American Type Culture Collection) and were grown at 37 °C, 5% CO_2_, and 100% humidity as described [41]. DSMZ and ATCC authenticate all cell lines by STR profiling and other methods before distribution. Total RNA and genomic DNA were isolated from all cell lines within three to eight passages after receipt as described previously [41, 42].

### Statistical Analyses

Statistical analyses were performed with R version 3.2.3 (“Wooden Christmas-Tree”), an open-source language and environment for statistical computing [43]. Differences between subgroups of patients or cell lines with respect to relative *CYP19A1* mRNA levels were analyzed by unpaired, two-sided *t* tests unless indicated otherwise. All *P* values shown are two-sided. *P* < 0.05 was considered statistically significant.

### Survival Analyses

Kaplan-Meier plots were computed with the R survival package [43]. *P* values to Kaplan-Meier curves were calculated by log-rank tests as described [44]. “Events” were defined as breast-cancer related death in the overall survival, and as affirmation of a distant metastasis, a second primary breast tumor, or a recurrent primary tumor in the disease-free survival. In the analyses of metastasis-free survival, the occurrence of a distant metastasis was considered as an event, and in the analyses of lung-, liver-, and bone-metastasis-free survival, only metastases to the respective target organ were considered. Among the 100 patients for whom expression of CYP19A1 could be determined, 57 events in the overall survival, 62 in the disease-free survival, and 52 in the metastasis-free survival were observed. The mean follow-up times were 7.6 ± 5.1 years for the overall survival (median, 6.7 years; range, 0–14.7 years), 5.5 ± 5.3 years for the disease-free survival (median, 3.4 years; range, 0–14.7 years), and 6.3 ± 5.4 years for the metastasis-free survival (median, 4.4 years; range, 0–14.7 years). For those patients who were still event-free at the end of follow-up, the mean follow-up times were 11.0 ± 4.8 years (median, 13.1 years; range, 0–14.7 years) for the overall survival, 10.1 ± 5.2 years (median, 12.8 years; range, 0–14.7 years) for the disease-free survival, and 9.9 ± 5.2 years for the metastasis-free survival (median, 12.8 years; range, 0–14.7 years). Details for all subgroups (ER positive and negative; CYP19A1 high and low; and rs10046 genotypes) and for all survival analyses are provided in Supplementary Tables 2 and 3.

## Results

### Association of *CYP19A1* mRNA Expression with rs10046 SNP Genotype and ER Status

**Fig. 1:**
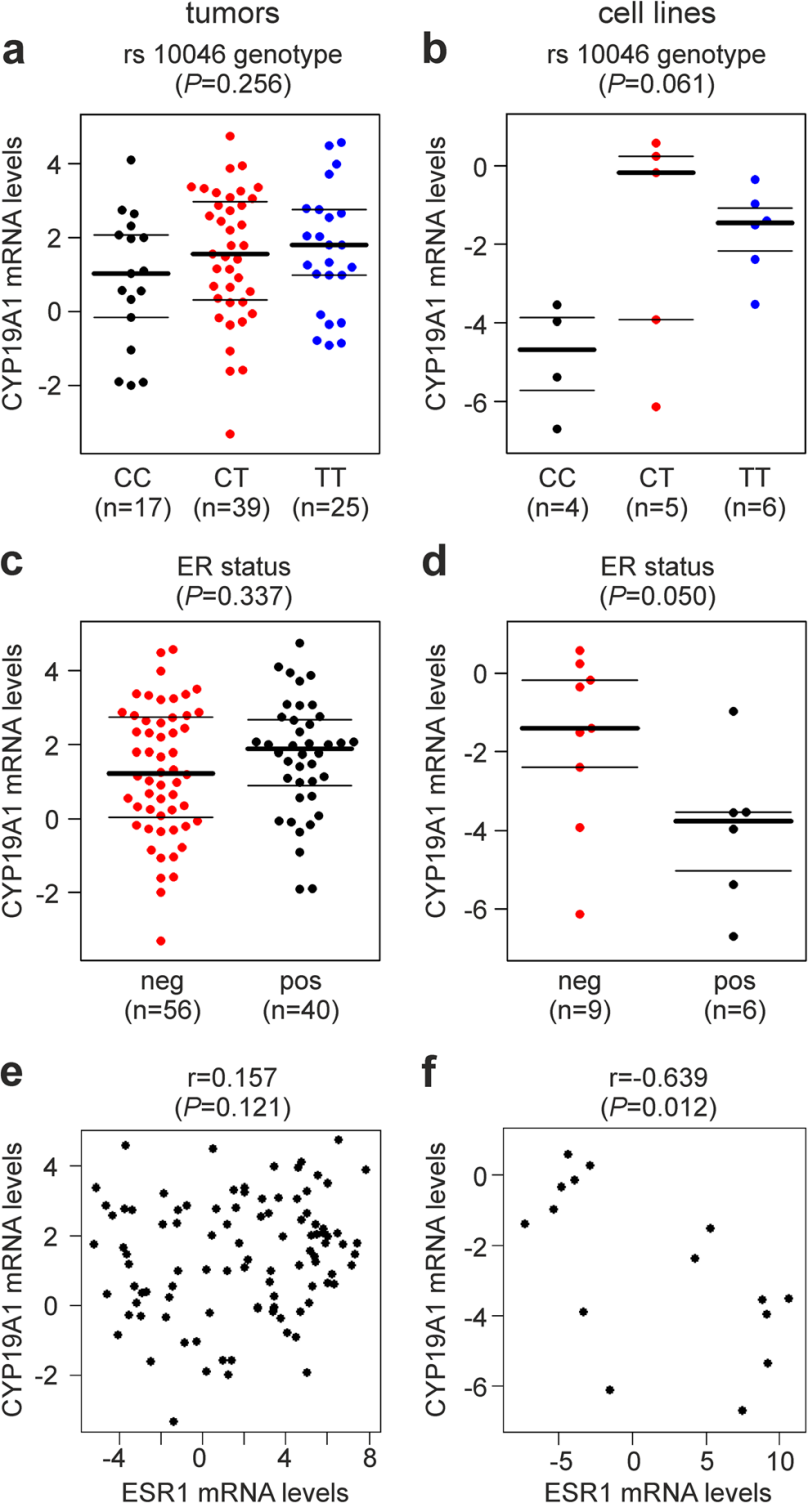
Association of *CYP19A1* mRNA expression with **a**, **b** rs10046 SNP genotype, **c**, **d** ER (estrogen receptor) status, and **e**, **f** *ESR1* (estrogen receptor 1) mRNA expression. Analyses are in human primary breast tumors (**a**, **c**, e) and in human breast cancer cell lines (**b**, **d**, **f**). CC, CT, and TT are the genotypes of SNP rs10046. The numbers of patients and cell lines in each group (*n*) are shown in parentheses. The *y*-axes in **a**–**f** show normalized relative *CYP19A1* mRNA levels (log(2) values). *P* values (*P*, in parentheses above each panel) were determined by **a**, **b** ANOVA, **c**, **d** unpaired, two-sided *t* tests, and **e**, **f** Spearman’s rank correlation. r, Spearman’s rho (rank correlation coefficient)

Genotypes of SNP rs10046 (CYP19 E10 c.+19C>T), located in the 3′UTR in exon 10 of the *CYP19A1* gene, were successfully determined in 119 breast cancer patients. The study population exhibited a frequency of 53.4% for the T-allele and was in Hardy-Weinberg equilibrium with respect to rs10046 genotypes (*P* = 0.52). Clinical characteristics and genotypes of these patients are shown in Supplementary Table 1. rs10046 was also genotyped in 15 human breast cancer cell lines, and genotype TT was detected in six (Cama1, Hcc1937, Hs578T, MDA-MB-231, MDA-MB-468, and ZR75-1), genotype CT in five (AU565, Hcc1143, MDA-MB-435, MDA-MB-453, and SKBR3), and genotype CC in four cell lines (BT474, Kpl1, MCF7, and T47D). *CYP19A1* mRNA expression levels were also determined for these 15 cell lines and 100 tumor samples. Overall, *CYP19A1* levels were not significantly associated with rs10046 genotypes in breast tumors or breast cancer cell lines (*P* = 0.256 and *P* = 0.061, respectively; ANOVA; Fig. 1a, b). However, mean *CYP19A1* mRNA levels in samples with the TT genotype were considerably higher than those in CC samples both in tumors (1.9-fold; *P* = 0.11, *t* test; Fig. 1a) and in cell lines (9.2-fold; *P* = 0.01; Fig. 1b).

Next, potential associations of *CYP19A1* expression with estrogen receptor (ER) status were investigated (Fig. 1c, d). Whereas no significant association was found in breast tumor samples (*P* = 0.337, *t* test; Fig. 1c), ER-positive breast cancer cell lines exhibited 5.1-fold lower mean *CYP19A1* mRNA levels than ER-negative ones (*P* = 0.050; Fig. 1d). Correlations of *CYP19A1* with *ESR1* (estrogen receptor 1) mRNA levels were assessed using Spearman’s rank correlation coefficient and visualized by scatterplots (Fig. 1e, f). No such correlation was observed in tumors (*r* = 0.157, *P* = 0.121; Fig. 1e). In contrast, a significant negative correlation of CYP19A1 and ESR1 levels was found in tumor cell lines (*r* = −0.639, *P* = 0.012; Fig. 1f).

### Analysis of *CYP19A1* Expression in Human Breast Cancer and Non-cancer Cell Lines

**Fig. 2:**
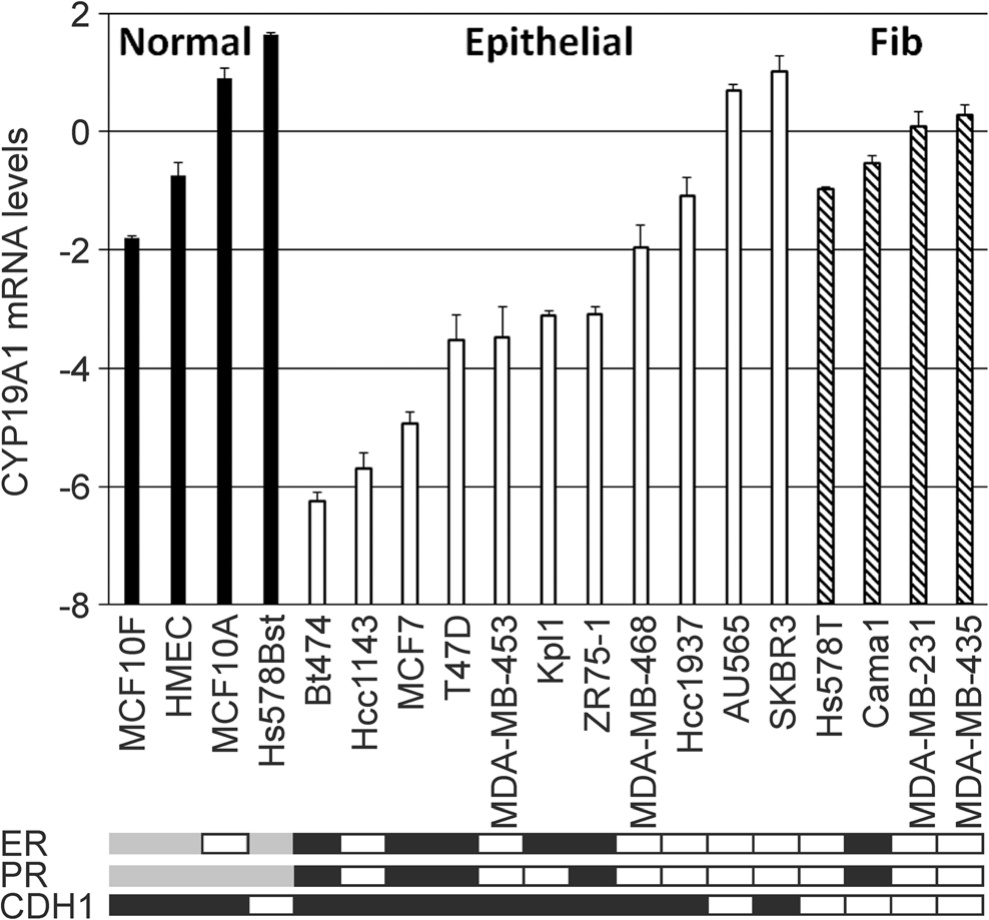
*CYP19A1* mRNA expression in human untransformed breast epithelial and breast cancer cell lines. The *y*-axis shows mean relative *CYP19A1* mRNA levels (log(2) values) of duplicate measurements. Expression levels of the indicated individual untransformed cells (Normal, black bars), breast cancer cell lines with an epithelial morphology (Epithelial, white bars), and breast cancer cell lines with a fibroblastoid morphology in culture (Fib, hatched bars) are shown. For normalization, the mean expression level of the four untransformed cell lines was set to unity (i.e., 2^0^ in graph), and the levels of individual cell lines are presented relative to those. Boxes underneath the bar graph indicate a positive (black) or negative (white) status of ER, PR, and CDH1 (E-cadherin). ER and PR status are based on [45, 46], and CDH1 status is based on determination of mRNA levels by qRT-PCR. Gray, status not available

We quantified *CYP19A1* mRNA expression levels in 15 human breast cancer cell lines and in four untransformed breast epithelial cell lines (Fig. 2). Breast cancer cell lines tended to have 4.5-fold lower mean *CYP19A1* expression levels compared to normal, untransformed breast epithelial cell lines (*P* = 0.06, *t* test). In an exploratory analysis, we found that this difference resulted mostly from cell lines with an epithelial morphology and a positive E-cadherin status (Fig. 2). Conversely, E-cadherin-negative tumor cell lines with a fibroblastoid morphology expressed *CYP19A1* at roughly the same level as untransformed cell lines. Accordingly, fibroblastoid breast cancer cell lines exhibited a 5.9-fold higher mean *CYP19A1* expression compared to cell lines with an epithelial phenotype (*P* = 0.006; Fig. 2). The ER-positive cell lines within this panel (BT474, Cama1, Kpl1, MCF7, T47D, and ZR75-1) all exhibited an epithelial morphology except Cama1. The five ER-positive epithelial cell lines were among those with the lowest *CYP19A1* expression (Figs. 1d and 2).

### Association of *CYP19A1* Expression with Clinical and Histopathological Characteristics in Primary Human Breast Tumor Tissue

**Fig. 3:**
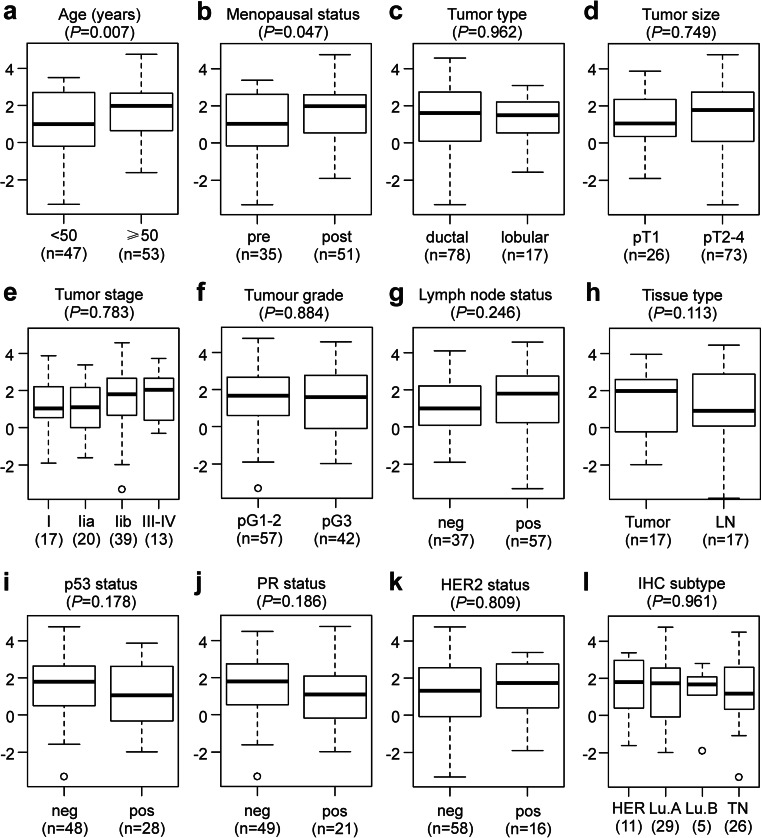
Association of *CYP19A1* mRNA expression with established clinical and histopathological parameters. Boxplots according to **a** age at breast cancer onset in years, **b** menopausal status, **c** tumor type, **d** tumor size according to TNM classification, **e** tumor stage, **f** tumor grade, **g** lymph node status, **h** in paired primary tumors and lymph node metastases (LN) of 17 patients, **i** p53 status, **j** progesterone receptor (PR) status, **k** HER2-status, and **l** molecular subtype, i.e., HER2-type (HER), Luminal A (Lu.A), Luminal B (Lu.B), and triple negative (TN). neg, negative; pos, positive. The numbers of patients in each group (n) are shown in parentheses. The *y*-axes show normalized relative *CYP19A1* mRNA levels (log(2) values). *P* values (*P*, in parentheses above each panel) were determined by **e**, **l** ANOVA; **h** paired, two-sided *t* tests; and unpaired, two-sided *t* tests in all other panels

Relative *CYP19A1* mRNA expression levels were quantified in primary tumor tissue samples of 100 breast cancer patients. Associations of *CYP19A1* expression with well-established clinical and histopathological characteristics of breast cancer were visualized with boxplots (Fig. 3). For 17 of these patients, one lymph node metastasis each was also analyzed, which exhibited 1.5-fold lower mean *CYP19A1* levels than their matched primary tumors; however, this difference was not significant (*P* = 0.113; Fig. 3h). Mean *CYP19A1* expression levels were found to be significantly elevated in breast tumor tissue samples of patients with an age at breast cancer onset ≥ 50 years (1.9-fold; *P* = 0.007; Fig. 3a) and in postmenopausal patients (1.7-fold; *P* = 0.047; Fig. 3b). In contrast, no significant association with *CYP19A1* expression was found in analyses of the tumor type, size, stage, and grade, as well as for lymph node status, p53-, PR-, HER2-status, and the molecular subtype (Fig. 3c–g, i–l), consistent with previous reports [13, 15, 19, 47–50]. Interestingly, a positive p53 status tended to be associated with both a lower expression of *CYP19A1* (Fig. 3i) and a higher frequency of the rs10046 CC genotype (Supplementary Table 1).

### Association of *CYP19A1* Expression with Breast Cancer Prognosis

**Fig. 4:**
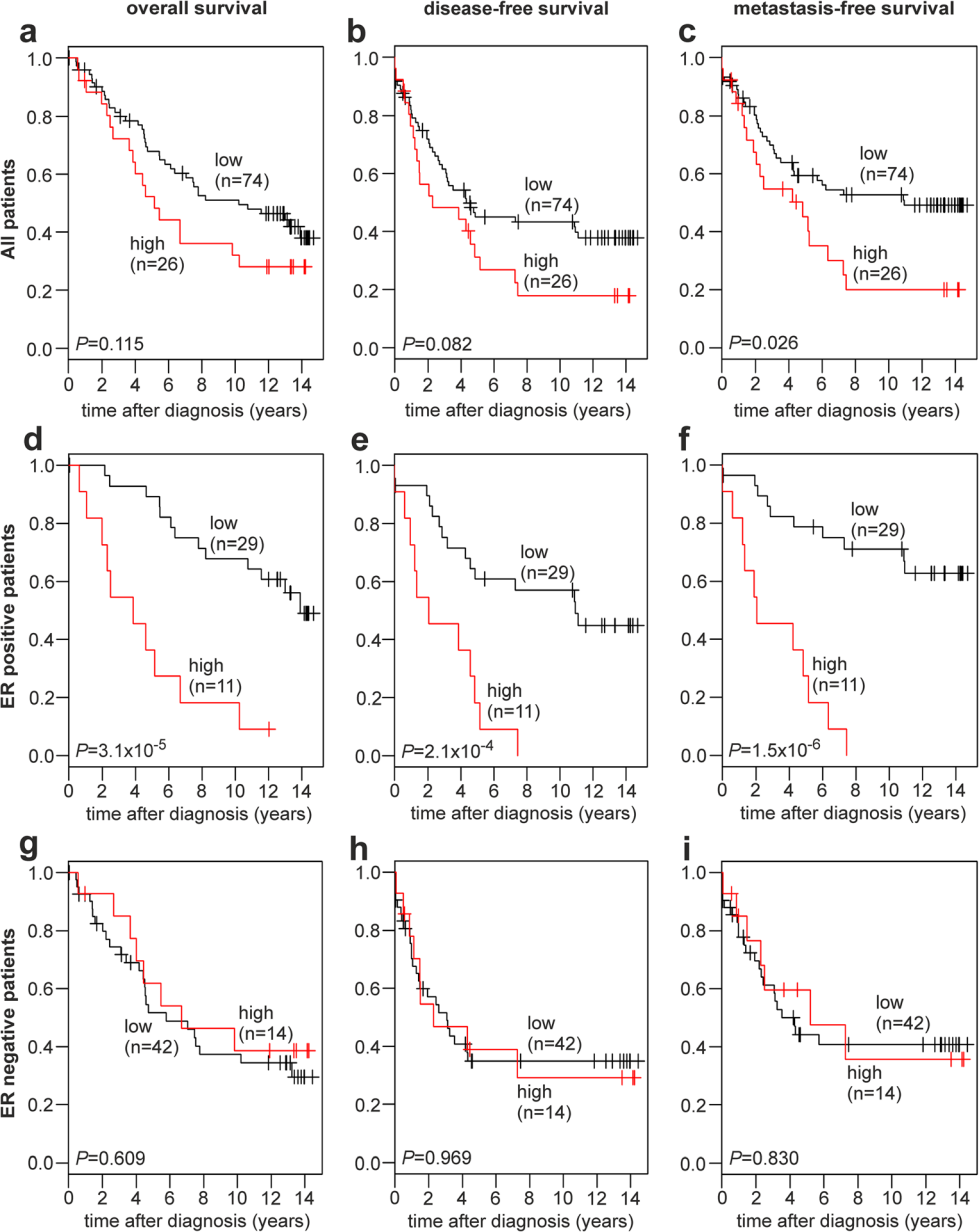
Association of *CYP19A1* mRNA expression with the survival of human breast cancer patients. Kaplan-Meier analyses of the overall survival (**a**, **d**, **g**), disease-free survival (**b**, **e**, **h**) and metastasis-free survival (**c**, **f**, **i**) in unselected patients (**a**–**c**; *n* = 100), ER-positive patients (**d**–**f**; *n* = 40), and ER-negative patients (**g**–**i**; *n* = 56) are shown. Patient subgroups with high and low *CYP19A1* expression and their numbers (*n*) are indicated in each panel. ER, estrogen receptor

Patients were divided into two subgroups based on *CYP19A1* expression by applying maximally selected log rank statistics to define a cutoff in unselected patients as described [44]. Subsequently, Kaplan-Meier analyses of the overall survival (OS), disease-free survival (DFS), and metastasis-free survival (MFS) were performed in unselected patients (Fig. 4a–c), in ER-positive patients (Fig. 4d–f), and in ER-negative patients (Fig. 4g–i). Unselected patients with a high *CYP19A1* expression exhibited a significantly decreased MFS (*P* = 0.026; Fig. 4c), as well as a trend towards a decreased OS (*P* = 0.115; Fig. 4a) and DFS (*P* = 0.082; Fig. 4b) compared to patients with a low CYP19A1 expression.

In ER-positive patients, highly significant differences between patients with high vs. low *CYP19A1* expression were observed with respect to OS (*P* = 3.1 × 10^−5^; Fig. 4d), DFS (*P* = 2.1 × 10^−4^; Fig. 4e), and MFS (*P* = 1.5 × 10^−6^; Fig. 4f). In each of these analyses, patients with a high *CYP19A1* expression exhibited a very poor survival. All ER-positive patients with a high *CYP19A1* expression (*n* = 11) progressed to metastasis and/or recurrent disease within less than 8 years after diagnosis (Fig. 4e, f). In contrast, ER-negative patients exhibited no difference in OS, DFS, or MFS between high and low *CYP19A1* expressers (Fig. 4g–i), indicating that the shorter OS, DFS, and MFS of unselected patients with high *CYP19A1* expression results exclusively from the ER-positive subgroup. Adjuvant tamoxifen therapy affected the association of *CYP19A1* expression with the survival of these patients more modestly than ER status (Supplementary Fig. 6).

In a hypothesis-generating analysis, metastases to the major target organs lung, liver, and bone were further examined (Supplementary Fig. 1; Supplementary Table 3). Unselected patients with high *CYP19A1* expression showed a trend towards decreased lung-MFS (*P* = 0.068) and bone-MFS (*P* = 0.072) compared to patients with low *CYP19A1* expression, whereas no association was found in the analysis of liver-MFS (*P* = 0.605; Supplementary Fig. 1). ER-positive patients with high *CYP19A1* expression exhibited a very poor survival free of lung metastases (*P* = 6.3 × 10^−7^) and bone metastases (*P* = 1.7 × 10^−8^) and a significant difference in liver-MFS (*P* = 0.003; Supplementary Fig. 1). Specifically, all uncensored ER-positive patients with a high *CYP19A1* expression (*n* = 11) had developed both lung and bone metastases within 10 years after diagnosis (Supplementary Fig. 1). *CYP19A1* expression was not associated with lung- and bone-MFS in ER-negative patients. In contrast to all other results of our survival analyses, high *CYP19A1* expression was associated with a good prognosis with respect to liver-MFS; no liver metastases were observed in this subgroup of 14 patients up to 14 years postdiagnosis (*P* = 0.043; Supplementary Fig. 1). However, it should be noted that these analyses are limited by the smaller number of events compared to the analyses of OS, DFS, and MFS (Supplementary Tables 2 and 3).

### Association of rs10046 SNP Genotypes with Breast Cancer Prognosis

The overall survival (OS), disease-free survival (DFS), and metastasis-free survival (MFS) associated with the three rs10046 genotypes CC, CT, and TT were compared in Kaplan-Meier analyses of unselected patients, ER-positive patients, ER-negative patients, premenopausal patients, and postmenopausal patients (Supplementary Figs. 2 and 4; Supplementary Table 2). No significant differences in any of these analyses were observed. Metastasis to the major target organs lung, liver, and bone was further examined (Supplementary Fig. 3; Supplementary Table 3). The survival analyses in Supplementary Figs. 2 and 3 revealed that patients with the TT genotype tended to have a better prognosis than CC patients if they were ER positive, but a poorer prognosis if they were ER negative. This was most pronounced in the metastases-free survival of ER-negative patients, where the TT genotype was associated with a considerably higher frequency of events than the CC genotype, and the CT genotype was intermediate (Supplementary Figs. 2 and 3; Supplementary Table 2). However, none of these trends was significant. Likewise, patients with the TT genotype tended to have a better prognosis than CC patients if they had received adjuvant tamoxifen therapy, but a poorer prognosis if they were not treated with tamoxifen (Supplementary Fig. 5).

## Discussion

Breast tumor cells and/or their surrounding stroma frequently express elevated levels of aromatase, producing sufficient local estrogen concentrations to sustain tumor cell proliferation and progression [6, 7]. Moreover, aromatase inhibitors are a current first line endocrine therapy for postmenopausal, hormone receptor-positive breast cancer [9, 10]. Accordingly, the potential impact of aromatase expression and genetic variants in *CYP19A1* on levels of circulating estrogen, breast cancer risk and prognosis, and response to endocrine therapy has been extensively studied [7, 30, 51, 52]. Among other *CYP19A1* variants, the rs10046 SNP has been associated with increased levels of circulating estrogen [3, 20–23] and an increased breast cancer risk in some studies [25, 28]. However, most studies, including the larger ones and meta-analyses, did not find an association of rs10046 with breast cancer risk [26, 27, 29–31].

We observed a very poor overall, disease-free, and metastasis-free survival of ER-positive breast cancer patients with a high *CYP19A1* expression. Whereas high levels of significance were obtained in ER-positive patients, aromatase expression was not significantly associated with survival in ER-negative patients. Since aromatase functions in the biosynthesis of estrogen, which exerts its breast cancer-promoting effects primarily via the estrogen receptor, these findings appear biologically plausible [5, 52, 53]. Moreover, since aromatase inhibitors prolong the disease-free and overall survival of ER-positive breast cancer, it seems reasonable that high aromatase expression has the opposite effect [8, 9]. A shortened disease-free survival of unselected breast cancer patients with a high *CYP19A1* mRNA expression has been reported previously [15]. Moreover, higher *CYP19A1* mRNA levels were significantly associated with the incidence of metastasis and local recurrence as well as breast cancer-related death [15]. Consistent with our results, aromatase IHC positivity was associated with a shortened overall survival in ER-positive but not ER-negative breast cancer [16].

However, other studies found no evidence for a significant association of aromatase mRNA, protein, or activity with prognosis in unselected [11–14, 17] or in ER-positive patients [19]. Intriguingly, low intra-tumoral *CYP19A1* mRNA expression was significantly associated with an increased locoregional recurrence rate in premenopausal patients younger than 40 years [18]. These authors suggested that, since estrogen represses the *CYP19A1* promoter, *CYP19A1* mRNA levels might be inversely correlated with the high circulating estrogen levels in premenopausal patients, consistent with the findings by us and others that CYP19A1 levels are lower in premenopausal patients and in patients younger than 50 years [14, 54]. Accordingly, high estrogen levels might be causal to both low *CYP19A1* expression and a high recurrence rate in these premenopausal patients [18]. Three studies have shown that in contrast to low expression, the complete absence of CYP19A1 protein or mRNA expression was associated with a poor prognosis [17, 50, 54]. These seemingly paradoxical results have been suggested to possibly be due to prior adjuvant or neoadjuvant treatment with aromatase inhibitors and/or tamoxifen or to reflect ongoing progression of the disease to a hormone-independent state [17, 50, 54]. We found that a low *CYP19A1* expression as well as the TT genotype of rs10046 tended to be associated with a favorable prognosis in patients with, but not those without adjuvant tamoxifen therapy (Supplementary Figs. 5 and 6). However, the number of ER-positive, untreated patients was too small to discern the potential effects of ER status and tamoxifen therapy.

In contrast to *CYP19A1* expression, we found no significant association of rs10046 genotypes with breast cancer prognosis in our analyses of the overall survival, disease-free survival, and metastasis-free survival in unselected, ER-positive, and ER-negative patients (Supplementary Figs. 2 and 3). Previous studies have also found either no association or an association in specific subgroups, specifically of the DFS in premenopausal patients [33–37]. In contrast, we did not find a significant association in premenopausal patients. This may in part be due to small numbers, since there were only 33 premenopausal patients with confirmed rs10046 genotypes in our study population (Supplementary Fig. 4).

Like other *CYP19A1* variants, the rs10046 SNP has been associated with circulating estrogen levels and the estradiol/testosterone ratio [3, 20–23]. Per T-allele of rs10046, the estradiol/testosterone ratio was found increased by ~ 10% [3, 23]. rs10046 is located in the 3′ UTR of *CYP19A1* and might regulate mRNA stability by miRNA binding and/or other mechanisms. Alternatively, rs10046 could be in strong linkage disequilibrium with one or more causal promoter SNPs rather than having a direct causal effect itself. In a recent systematic analysis, one of several *CYP19A1* SNPs other than rs10046, but in linkage disequilibrium with it, was demonstrated to indeed be causal to the elevated estrogen levels [23]. One study has reported that the T-allele of rs10046 is associated with elevated levels of *CYP19A1* mRNA in breast tumors [25]. However, that study used a now out-dated method of RNA quantification and did not report actual mRNA levels. Instead, the samples were grouped according to *CYP19A1* levels above and below median and subjected to chi-square tests [25]. We are not aware of any additional, more recent analyses of the association of rs10046 genotype with aromatase expression in breast cancer. Here, we found no evidence for a significant association of *CYP19A1* levels with rs10046 genotypes in breast tumors or breast cancer cell lines (Fig. 1). However, *CYP19A1* levels were clearly elevated in tumors and cell lines with the TT genotype compared to CC samples, in line with the increased circulating estrogen levels associated with the T-allele.

A trend towards a positive correlation between aromatase and ER expression has been reported [14, 15, 49, 50, 55, 56], whereas others found no evidence for a correlation [18, 54, 57–59] or even a non-significant inverse correlation [60]. We also found non-significant positive correlations of *CYP19A1* mRNA expression with ER status and with *ESR1* mRNA expression in breast tumors. In contrast, we found significant inverse correlations in breast cancer cell lines (Fig. 1). However, this in vitro result may be confounded by the fact that five out of six ER-positive cell lines were of epithelial morphology, which was associated with a much lower *CYP19A1* expression than the fibroblastoid morphology (Fig. 2). Moreover, it may be explained by culture conditions and by the absence of stromal cells from in vitro culture, which are engaged in extensive gene-regulatory signaling crosstalk with tumor cells in situ and are also a substantial source of intra-tumoral aromatase expression [1, 7].

A possible limitation of the present study relates to the size of our study population. In particular, the number of patients in which both *CYP19A1* expression and rs10046 genotype were successfully determined was only 81, which may explain why we were unable to replicate the significantly elevated *CYP19A1* mRNA levels in TT patients in a previous study of 99 patients [25]. On the other hand, survival analyses represent the main part of our study, in which the statistical power is determined by the number of events, ameliorating the limitations of our study population. Since other *CYP19A1* polymorphisms which are even located within promoter elements were found either not significantly associated [61], or only nominally associated with aromatase expression in non-malignant tissue [23], we believe that the next urgent issue is to convincingly demonstrate the mechanism by which *CYP19A1* variants cause the elevated levels of circulating estrogen, be it by altering the expression or the activity of aromatase.

In summary, we report that *CYP19A1* mRNA expression is a powerful prognostic marker in ER-positive but not ER-negative breast cancer, is significantly elevated in postmenopausal patients and in patients older than 50 years, and tends to positively correlate with rs10046 risk allele, ER status, and *ESR1* mRNA expression.

## Electronic Supplementary Material

ESM 1 (PDF 1675 kb)
