# Supplementary material for: Elevated Aromatase (CYP19A1) Expression Is Associated with a Poor Survival of Patients with Estrogen Receptor Positive Breast Cancer
Source: Horm Cancer. 2018 Jan 23;9(2):128–38. doi: 10.1007/s12672-017-0317-2 (PMC5862917; doi:10.1007/s12672-017-0317-2)
Supplement: Supplementary file 1 — (PDF 1675 kb) [file 12672_2017_317_MOESM1_ESM.pdf]

Supplementary Material

Elevated aromatase *(CYP19A1)* expression is associated with a poor survival of patients with estrogen receptor positive breast cancer

Hormones and Cancer

Andrea Friesenhengst, Tamara Pribitzer-Winner, Heidi Miedl, Katharina Pröstling, and Martin Schreiber

*Corresponding author: Martin Schreiber, Ph.D,

Department of Obstetrics & Gynecology, Medical University of Vienna,
Waehringer Guertel 18-20/5Q, 1090 Vienna, Austria

E-mail address: martin.schreiber@muv.ac.at

Supplementary Table 1 Representation of the indicated clinical characteristics in human breast cancer patients with SNP rs10046 genotypes CC, CT and TT

		Sum	CC	CT	TT
All patients		119	28 (23.5%)	55 (46.2%)	36 (30.3%)
Age	<50	44	13 (29.5%)	19 (43.2%)	12 (27.3%)
	≥50	75	15 (20.0%)	36 (48.0%)	24 (32.0%)
Menopausal status	pre	33	9 (27.3%)	14 (42.4%)	10 (30.3%)
	post	67	15 (20.9%)	30 (44.8%)	23 (34.3%)
	na	19	5	11	3
Tumor size	pT1	37	9 (24.3%)	15 (40.5%)	13 (35.1%)
	pT2-4	80	18 (22.5%)	39 (48.8%)	23 (28.8%)
	na	2	1	1	0
Tumor type	ductal	83	17 (20.5%)	43 (51.8%)	23 (27.7%)
	lobular	32	10 (31.3%)	10 (31.3%)	12 (37.5%)
	na	4	1	2	1
Stage	1	24	5 (20.8%)	10 (41.7%)	9 (37.5%)
	2-4	84	21 (25.0%)	38 (45.2%)	25 (29.8%)
	na	11	2	7	2
Grade	pG1-2	70	15 (21.4%)	35 (50.0%)	20 (28.6%)
	pG3	47	12 (25.5%)	19 (40.4%)	16 (34.0%)
	na	2	1	1	0
Lymph node status	pN0	41	10 (24.4%)	18 (43.9%)	13 (31.7%)
	pN+	70	17 (24.3%)	33 (47.1%)	20 (28.6%)
	na	8	1	4	3
ER status	pos	52	12 (23.1%)	21 (40.4%)	19 (36.5%)
	neg	61	13 (21.3%)	31 (50.8%)	17 (27.9%)
	na	6	3	3	0
PR status	pos	24	5 (20.8%)	12 (50.0%)	7 (29.2%)
	neg	60	13 (21.7%)	29 (48.3%)	18 (30.0%)
	na	35	10	14	11
HER2 status	pos	17	3 (17.6%)	9 (52.9%)	5 (29.4%)
	neg	73	16 (21.9%)	35 (47.9%)	22 (30.1%)
	na	29	9	11	9
p53 status	pos	33	10 (30.3%)	16 (48.5%)	7 (21.2%)
	neg	58	9 (15.5%)	28 (48.3%)	21 (36.2%)
	na	28	9	11	8
Tumor Subtype	HER2 type	12	2 (16.7%)	6 (50.0%)	4 (33.3%)
	Luminal	42	9 (21.4%)	21 (50.0%)	12 (28.6%)
	Triple neg	31	8 (25.8%)	16 (51.6%)	7 (22.6%)
	na	34	9	12	13

Supplementary Table 2 Details of the analyses of the overall, disease-free and metastasis-free survival in the indicated patient subpopulations

		All patients		CYP19A1 high		CYP19A1 low		CC (rs10046)		CT (rs10046)		TT (rs10046)	
Overall Survival		All	event-free	All	event-free	All	event-free	All	event-free	All	event-free	All	event-free
All patients	number (n)	100	43	26	8	74	35	28	13	55	22	36	14
	events (n)	57	0	18	0	39	0	15	0	33	0	22	0
	mean (y)	7,55	10,96	6,53	11,68	7,92	10,83	6,46	7,63	6,99	10,54	6,63	10,36
	median (y)	6,70	13,13	4,88	13,33	7,49	13,13	5,40	12,55	5,15	13,03	5,62	13,54
	range (y)	0-14.7	0-14.7	0.6-14.2	1.0-14.2	0-14.7	0-14.7	0-16.6	0-16.6	0-14.4	0-14.4	0-14.3	0-14.3
ER pos patients	number (n)	40	17	11	1	29	16	12	3	21	8	19	8
	events (n)	23	0	10	0	13	0	9	0	13	0	11	0
	mean (y)	8,81	12,68	4,65	12,04	10,39	12,72	8,01	12,83	7,11	10,50	6,90	10,46
	median (y)	10,50	13,32	3,86	12,04	12,55	13,33	9,23	12,58	5,48	12,96	6,12	13,75
	range (y)	0-14.7	0-14.7	0.6-12.0	12.0	0-14.7	0-14.7	1.1-13.4	2.6-13.4	0-14.4	0-14.4	0-14.3	0-14.3
ER neg patients	number (n)	56	23	14	6	42	17	13	8	31	11	17	6
	events (n)	33	0	8	0	25	0	5	0	20	0	11	0
	mean (y)	6,55	9,70	7,53	11,35	6,26	9,20	6,52	7,58	6,29	9,78	6,32	10,23
	median (y)	4,56	12,88	6,08	13,43	4,49	12,88	4,43	7,27	4,52	12,93	4,74	13,19
	range (y)	0-14.5	0-14.5	0.6-14.2	1.0-14.2	0-14.5	0-14.5	0-16.6	0-16.6	0-14.4	0-14.4	0.4-14.2	3.1-14.2
Disease-free survival													
All patients	number (n)	100	38	26	6	74	32	28	14	55	24	36	16
	events (n)	62	0	20	0	42	0	14	0	31	0	20	0
	mean (y)	5,48	10,06	4,29	10,03	5,90	10,07	4,94	7,20	5,31	8,91	5,67	9,13
	median (y)	3,39	12,82	2,18	13,40	3,86	12,63	2,25	9,52	3,14	12,53	3,76	12,08
	range (y)	0-14.7	0-14.7	0-14.2	0.6-14.2	0-14.7	0-14.7	0-14.5	0-14.5	0-14.4	0-14.4	0-14.3	0-14.3
ER pos patients	number (n)	40	14	11	0	29	14	12	4	21	9	19	9
	events (n)	26	0	11	0	15	0	8	0	12	0	10	0
	mean (y)	6,67	11,85	2,91		8,10	11,85	6,42	12,31	6,21	8,61	5,84	8,37
	median (y)	4,86	13,33	2,06		10,77	13,33	4,86	12,57	4,84	12,51	5,43	11,56
	range (y)	0-14.7	0-14.7	0-7.5		0-14.7	0-14.7	0-13.4	0.8-13.4	0-14.4	0-14.4	0-14.3	0-14.3
ER neg patients	number (n)	56	22	14	5	42	17	13	7	31	12	17	7
	events (n)	34	0	9	0	25	0	6	0	19	0	10	0
	mean (y)	4,44	8,68	4,73	9,37	4,35	8,47	4,73	7,36	3,91	8,00	5,47	10,11
	median (y)	2,11	12,20	1,89	13,49	2,18	11,85	1,67	8,27	2,29	8,22	3,25	12,60
	range (y)	0-14.5	0-14.5	0.1-14.2	0.6-14.2	0-14.5	0-14.5	0-14.5	0-14.5	0-14.4	0-14.4	0-14.2	0.5-14.2
Metastasis-free survival													
All patients	number (n)	100	48	26	8	74	40	28	16	55	28	36	19
	events (n)	52	0	18	0	34	0	12	0	27	0	17	0
	mean (y)	6,30	9,91	4,60	8,10	6,90	10,27	5,66	7,55	5,75	8,31	6,02	9,23
	median (y)	4,38	12,80	3,08	8,88	5,03	12,80	3,86	9,52	3,68	12,18	3,76	12,60
	range (y)	0-14.7	0-14.7	0-14.2	0.6-14.2	0-14.7	0-14.7	0-16.6	0-16.6	0-14.4	0-14.4	0-14.3	0-14.3
ER pos patients	number (n)	40	19	11	0	29	19	12	4	21	9	19	12
	events (n)	21	0	11		10	0	8	0	12	0	7	0
	mean (y)	7,78	11,95	3,19		9,52	11,95	6,78	12,31	6,38	8,61	6,41	8,72
	median (y)	7,37	13,31	2,06		11,57	13,31	5,58	12,57	5,45	12,51	5,57	12,12
	range (y)	0-14.7	0-14.7	0-7.5		0-14.7	0-14.7	0-13.4	0.8-13.4	0-14.4	0-14.4	0-14.3	0-14.3
ER neg patients	number (n)	56	27	14	7	42	20	13	9	31	16	17	7
	events (n)	29	0	7	0	22	0	4	0	15	0	10	0
	mean (y)	5,08	8,25	5,08	7,36	5,08	8,57	5,94	7,95	4,58	7,19	5,57	10,12
	median (y)	3,11	11,85	3,08	4,45	3,11	12,20	3,46	8,27	2,93	4,57	3,25	12,60
	range (y)	0-14.5	0-14.5	0-14.2	0.6-14.2	0-14.5	0-14.5	0-16.6	0-16.6	0-14.4	0-14.4	0-14.2	0.5-14.2

All, all patients in the indicated analysis; event-free, those patients without an event in the indicated analysis; ER, estrogen receptor; pos, positive; neg, negative. The number of patients (n), the number of events (n), as well as the mean, median, and range for the follow-up time in years (y) is indicated in each analysis and subgroup

Supplementary Table 3 Details of the analyses of the survival free of lung, liver and bone metastasis in the indicated patient subpopulations

		All patients		CYP19A1 high		CYP19A1 low		CC (rs10046)		CT (rs10046)		TT (rs10046)	
Lung metastasis-free survival		All	event-free	All	event-free	All	event-free	All	event-free	All	event-free	All	event-free
All patients	number (n)	100	86	26	20	74	66	28	24	55	49	36	30
	events (n)	14	0	6	0	8	0	4	0	6	0	6	0
	mean (y)	7,33	7,72	6,16	6,41	7,74	8,11	6,37	6,27	6,77	7,10	6,44	7,09
	median (y)	6,07	6,94	4,34	4,23	6,94	7,66	5,22	3,95	4,60	5,16	5,12	6,04
	range (y)	0-14.7	0-14.7	0.6-14.2	0.6-14.2	0-14.7	0-14.7	0-16.6	0-16.6	0-14.4	0-14.4	0-14.3	0-14.3
ER pos patients	number (n)	40	34	11	7	29	27	12	9	21	19	19	18
	events (n)	6	0	4	0	2	0	3	0	2	0	1	0
	mean (y)	8,66	8,81	4,30	2,50	10,31	10,45	7,79	7,39	7,08	7,27	6,90	6,91
	median (y)	9,62	11,17	3,86	2,30	12,52	12,55	9,13	8,22	5,49	5,49	6,13	6,04
	range (y)	0-14.7	0-14.7	0.6-10.0	0.6-5.2	0-14.7	0-14.7	1.1-13.4	1.1-13.4	0-14.4	0-14.4	0-14.3	0-14.3
ER neg patients	number (n)	56	48	14	12	42	36	13	12	31	27	17	12
	events (n)	8	0	2	0	6	0	1	0	4	0	5	0
	mean (y)	6,27	6,85	7,12	8,11	5,99	6,43	6,52	6,99	5,92	6,28	5,91	7,37
	median (y)	4,44	4,57	5,58	8,28	4,31	4,53	4,43	5,74	4,01	4,20	3,25	6,25
	range (y)	0-14.5	0-14.5	0.6-14.2	0.6-14.2	0-14.5	0-14.5	0-16.6	0-16.6	0-14.4	0-14.4	0.5-14.3	0.5-14.3
Liver metastasis-free survival													
All patients	number (n)	100	83	26	23	74	60	28	23	55	48	36	30
	events (n)	17	0	3	0	14	0	5	0	7	0	6	0
	mean (y)	7,39	8,03	6,33	6,55	7,76	8,60	6,24	6,21	6,87	7,18	6,47	7,36
	median (y)	6,54	7,53	4,41	4,45	7,49	11,17	4,92	3,46	4,77	4,96	5,22	6,28
	range (y)	0-14.7	0-14.7	0-14.2	0-14.2	0-14.7	0-14.7	0-16.6	0-16.6	0-14.4	0-14.4	0-14.3	0-14.3
ER pos patients	number (n)	40	35	11	8	29	27	12	9	21	17	19	18
	events (n)	5	0	3	0	2	0	3	0	4	0	1	0
	mean (y)	8,68	9,10	4,50	4,46	10,27	10,48	7,87	7,73	6,92	7,57	6,87	7,25
	median (y)	10,14	11,57	3,86	3,19	12,52	12,55	8,86	8,22	5,45	6,71	6,13	6,28
	range (y)	0-14.7	0-14.7	0-12.0	1.1-12.0	0-14.7	0-14.7	1.1-13.4	1.1-13.4	0-14.4	0-14.4	0-14.3	0-14.3
ER neg patients	number (n)	56	45	14	14	42	31	13	11	31	28	17	12
	events (n)	11	0	0	0	11	0	2	0	3	0	5	0
	mean (y)	6,37	7,11	7,26	7,26	6,07	7,03	6,18	6,67	6,21	6,27	6,03	7,52
	median (y)	4,53	4,60	5,58	5,58	4,53	4,60	3,46	3,46	4,53	4,36	4,45	5,72
	range (y)	0-14.5	0-14.5	0.6-14.3	0.6-14.3	0-14.5	0-14.5	0-16.6	0-16.6	0-14.4	0-14.4	0-14.3	0.5-14.3
Bone metastasis-free survival													
All patients	number (n)	100	70	26	15	74	55	28	21	55	39	36	23
	events (n)	30	0	11	0	19	0	7	0	16	0	13	0
	mean (y)	6,97	8,53	5,50	6,93	7,48	8,97	5,90	6,65	6,38	7,61	6,26	8,16
	median (y)	5,33	10,31	4,53	4,61	6,60	11,85	3,89	6,37	4,60	5,49	4,36	11,31
	range (y)	0-14.7	0-14.7	0.1-14.2	0.6-14.2	0-14.7	0-14.7	0-16.6	0-16.6	0-14.4	0-14.4	0-14.3	0-14.3
ER pos patients	number (n)	40	27	11	2	29	25	12	7	21	14	19	13
	events (n)	13	0	9	0	4	0	5	0	7	0	6	0
	mean (y)	8,27	10,06	3,54	2,62	10,07	10,65	7,29	9,47	6,77	7,73	6,50	8,09
	median (y)	9,03	12,52	2,06	2,62	12,01	12,55	7,30	10,77	5,49	6,11	5,94	11,57
	range (y)	0-14.7	0-14.7	0.6-9.8	0.6-4.6	0-14.7	0-14.7	0-13.4	2.5-13.4	0-14.4	0-14.4	0-14.3	0-14.3
ER neg patients	number (n)	56	39	14	12	42	27	13	11	31	22	17	10
	events (n)	17	0	2	0	15	0	2	0	9	0	7	0
	mean (y)	5,90	7,44	6,48	7,12	5,71	7,59	5,99	6,67	5,43	6,75	6,00	8,25
	median (y)	4,23	5,46	4,83	4,95	3,92	7,46	3,46	3,46	3,68	4,53	4,27	8,38
	range (y)	0-14.5	0-14.5	0.1-14.2	0.6-14.2	0-14.5	0-14.5	0-16.6	0-16.6	0-14.4	0-14.4	0-14.2	0.5-14.2

All, all patients in the indicated analysis; event-free, those patients without an event in the indicated analysis; ER, estrogen receptor; pos, positive; neg, negative. The number of patients (n), the number of events (n), as well as the mean, median, and range for the follow-up time in years (y) is indicated in each analysis and subgroup

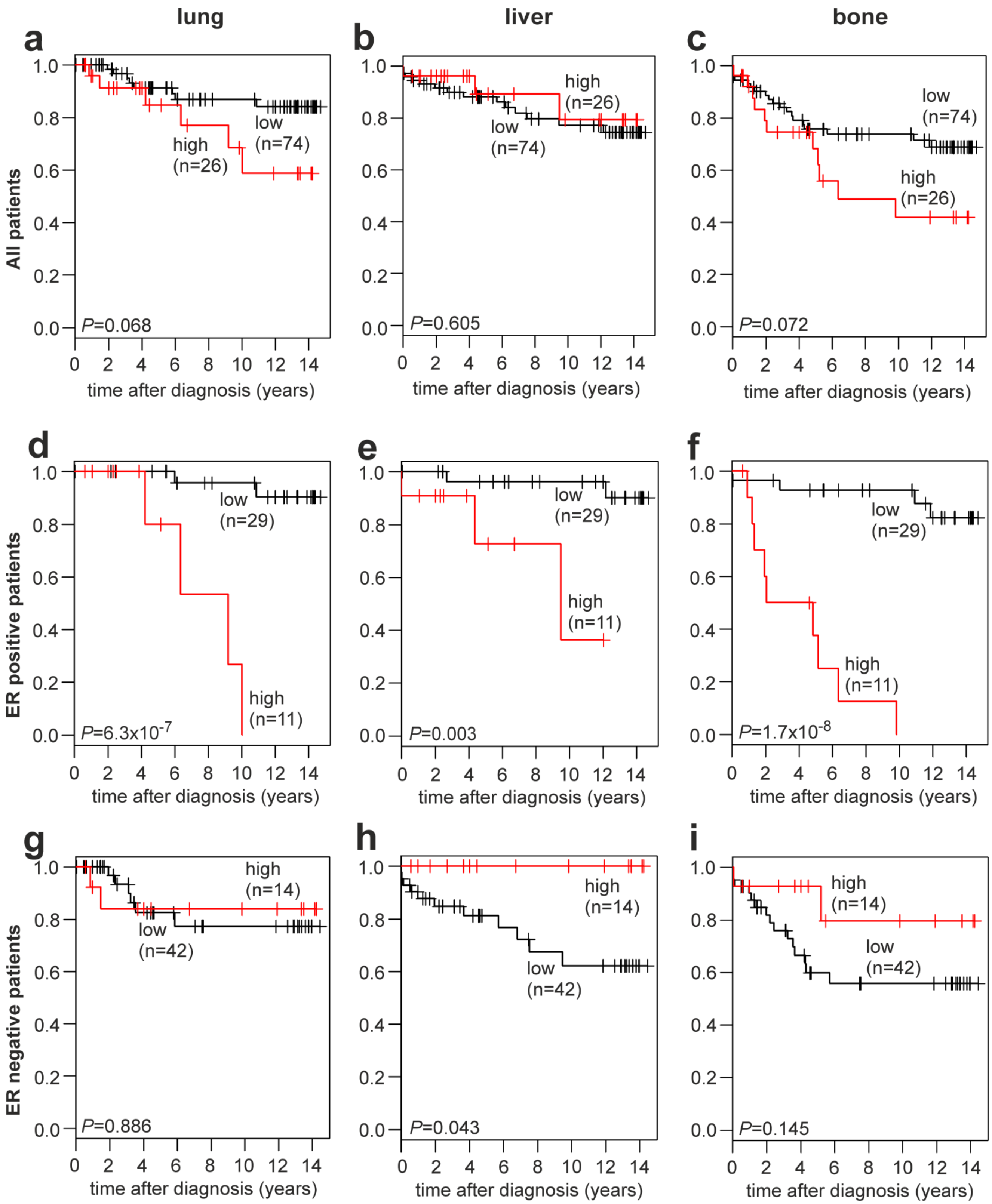

Supplementary Fig. 1 Association of *CYP19A1* mRNA expression with the metastasis-free survival of human breast cancer patients. Kaplan-Meier analyses of the lung metastasis-free survival (a, d, g), liver metastasis-free survival (b, e, h) and bone metastasis-free survival (c, f, i) in unselected patients (a-c; n=100), ER-positive patients (d-f; n=40) and ER-negative patients (g-i; n=56) are shown. Patient subgroups with high and low *CYP19A1* expression and their numbers (n) are indicated in each panel. ER, estrogen receptor

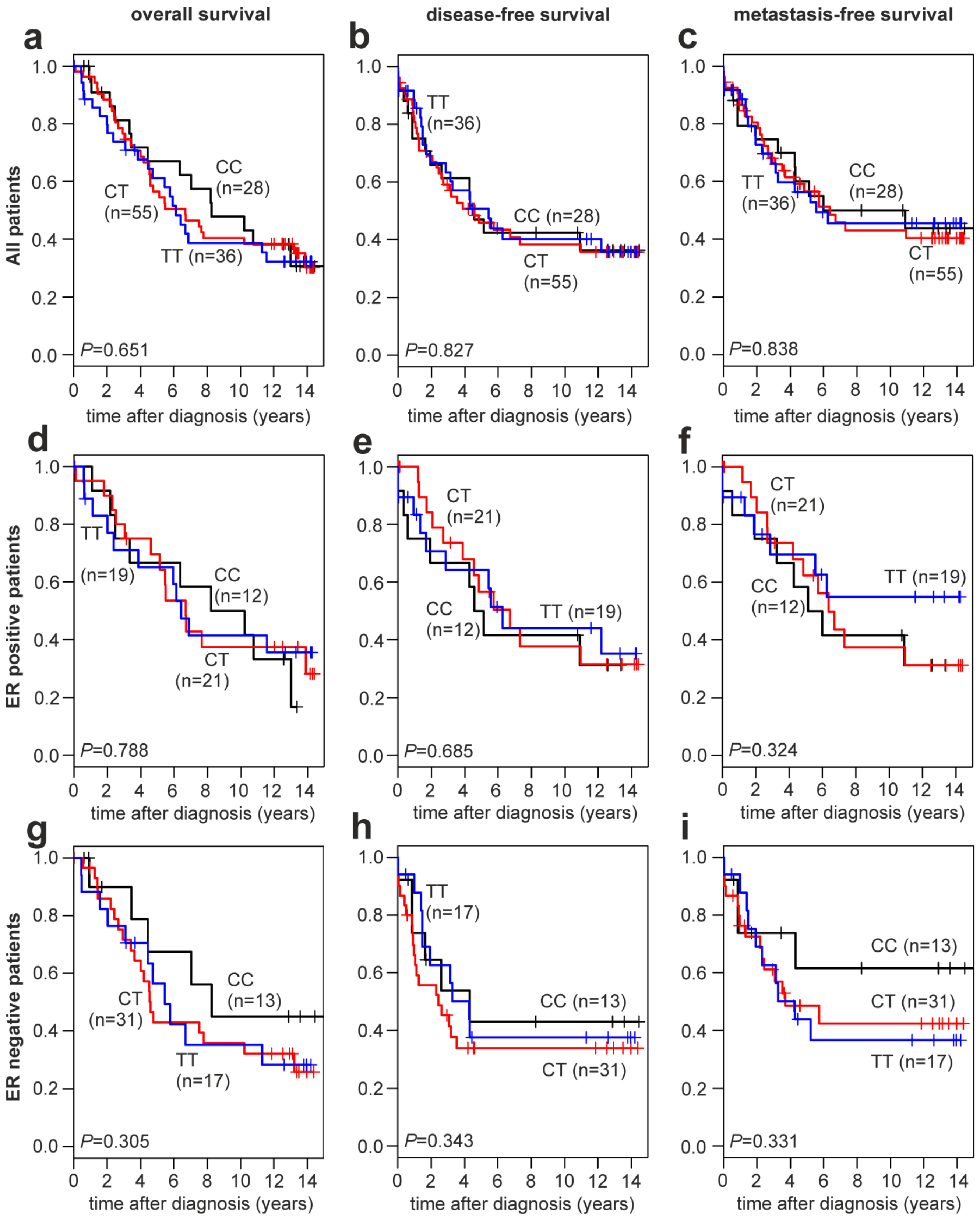

Supplementary Fig. 2 Association of *CYP19A1* rs10046 genotypes with the survival of human breast cancer patients. Kaplan-Meier analyses of the overall survival (a, d, g), disease-free survival (b, e, h) and metastasis-free survival (c, f, i) in unselected patients (a-c; n=119), ER positive patients (d-f; n=52) and ER negative patients (g-i; n=61) are shown. Patient subgroups with rs10046 genotypes CC, CT and TT as well as their numbers (n) are indicated in each panel. ER, estrogen receptor

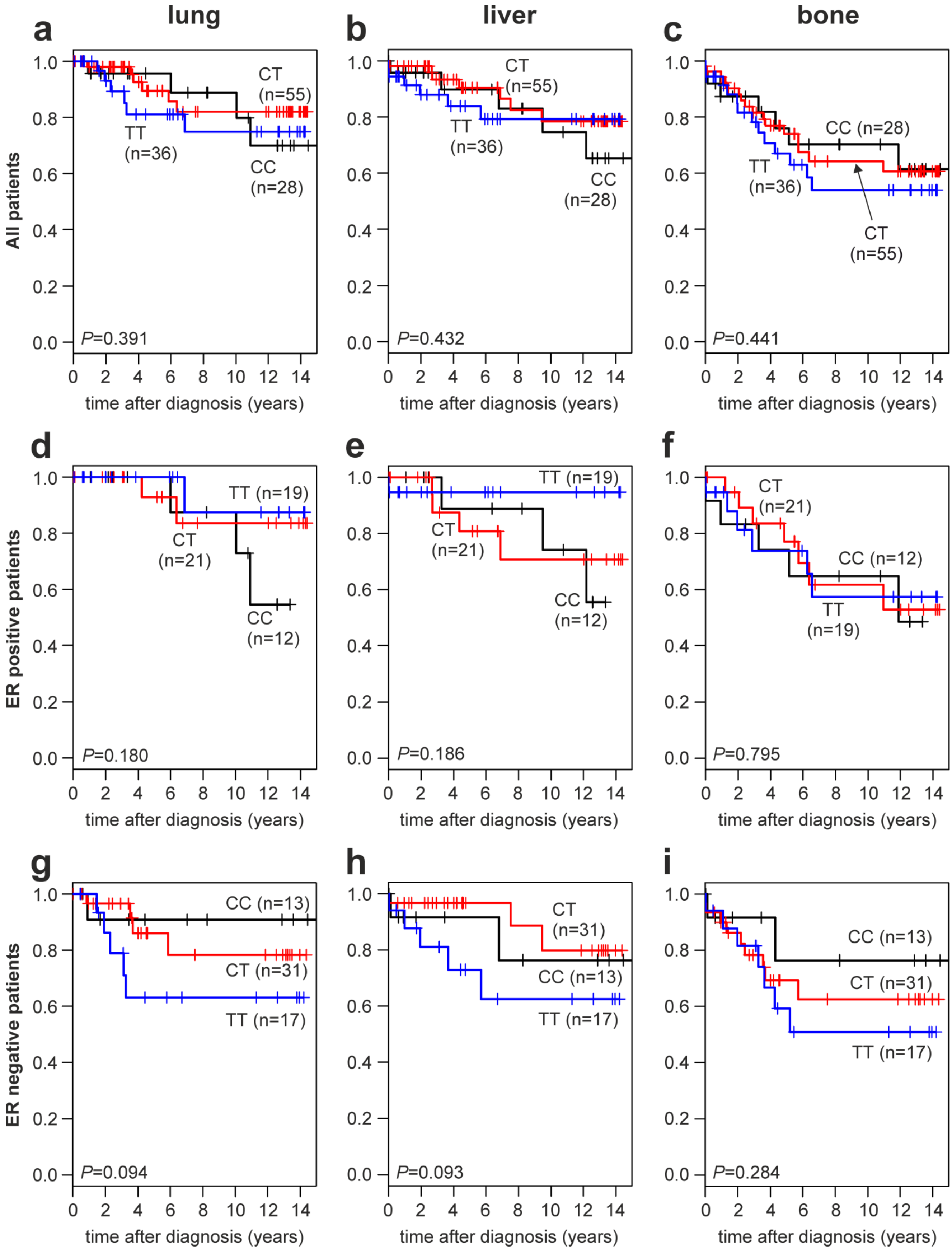

Supplementary Fig. 3 Association of *CYP19A1* rs10046 genotypes with the metastasis-free survival of human breast cancer patients. Kaplan-Meier analyses of the lung metastasis-free survival (a, d, g), liver metastasis-free survival (b, e, h) and bone metastasis-free survival (c, f, i) in unselected patients (a-c; n=119), ER positive patients (d-f; n=52) and ER negative patients (g-i; n=61) are shown. Patient subgroups with rs10046 genotypes CC, CT and TT as well as their numbers (n) are indicated in each panel. ER, estrogen receptor

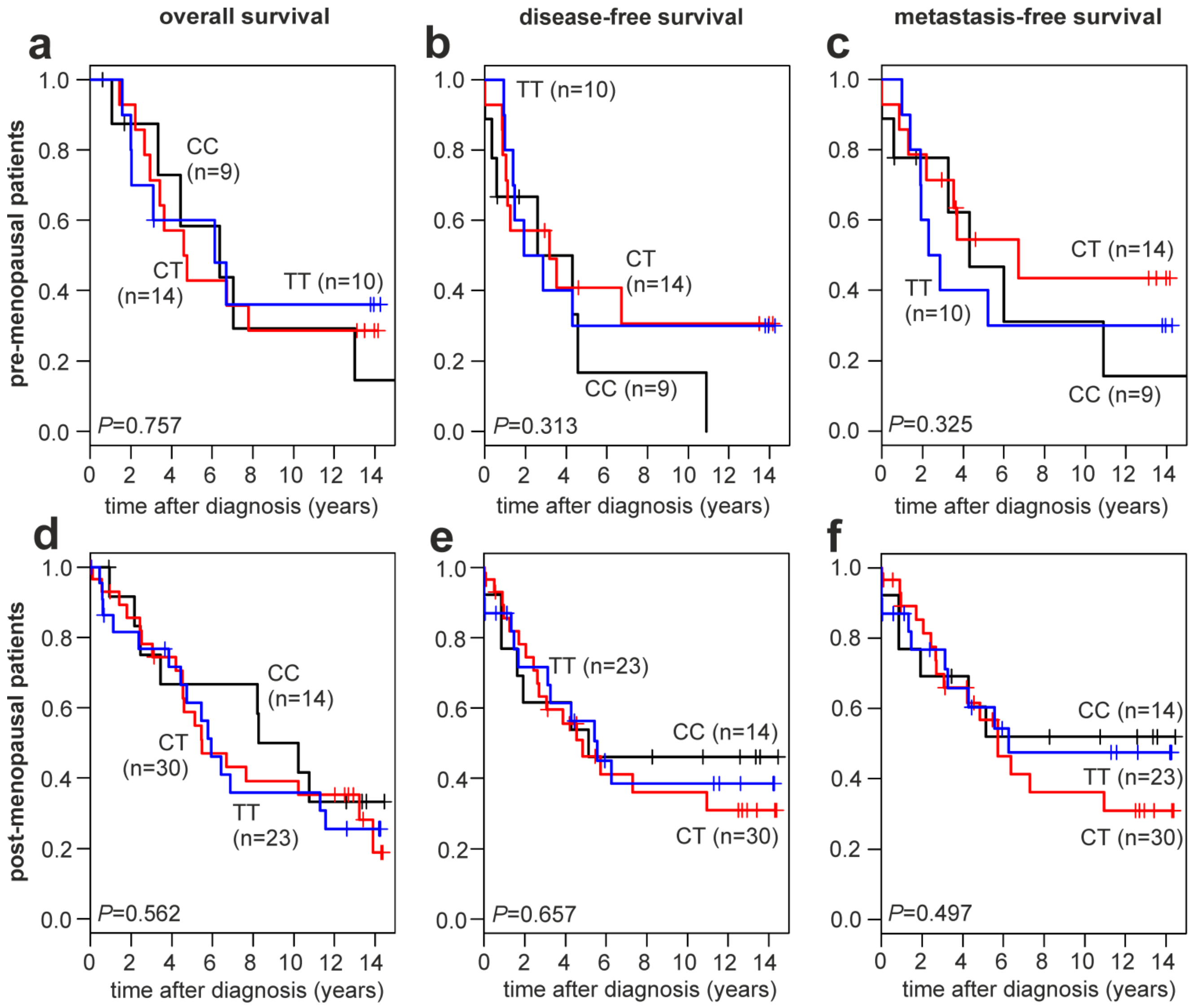

Supplementary Fig. 4 Association of *CYP19A1* rs10046 genotypes with the survival of human breast cancer patients. Kaplan-Meier analyses of the overall survival (a, d), disease-free survival (b, e) and metastasis-free survival (c, f) in pre-menopausal patients (a-c; n=33) and post-menopausal patients (d-f; n=67) are shown. Patient subgroups with rs10046 genotypes CC, CT and TT as well as their numbers (n) are indicated in each panel.

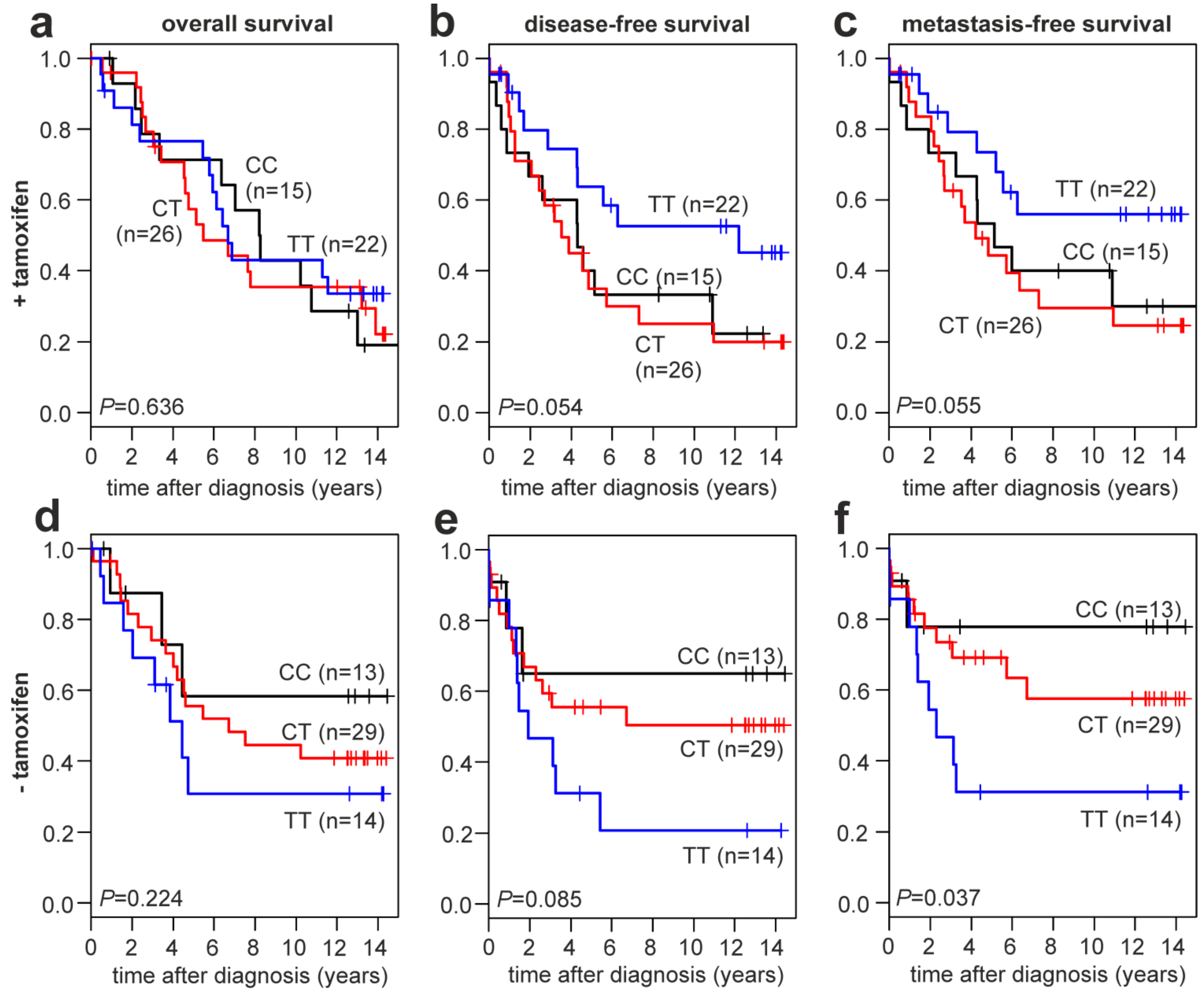

Supplementary Fig. 5 Association of *CYP19A1* rs10046 genotypes with the survival of human breast cancer patients. Kaplan-Meier analyses of the overall survival (a, d), disease-free survival (b, e) and metastasis-free survival (c, f) in patients treated with tamoxifen (a-c; n=63) and patients not treated with tamoxifen (d-f; n=56) are shown. Patient subgroups with rs10046 genotypes CC, CT and TT as well as their numbers (n) are indicated in each panel.

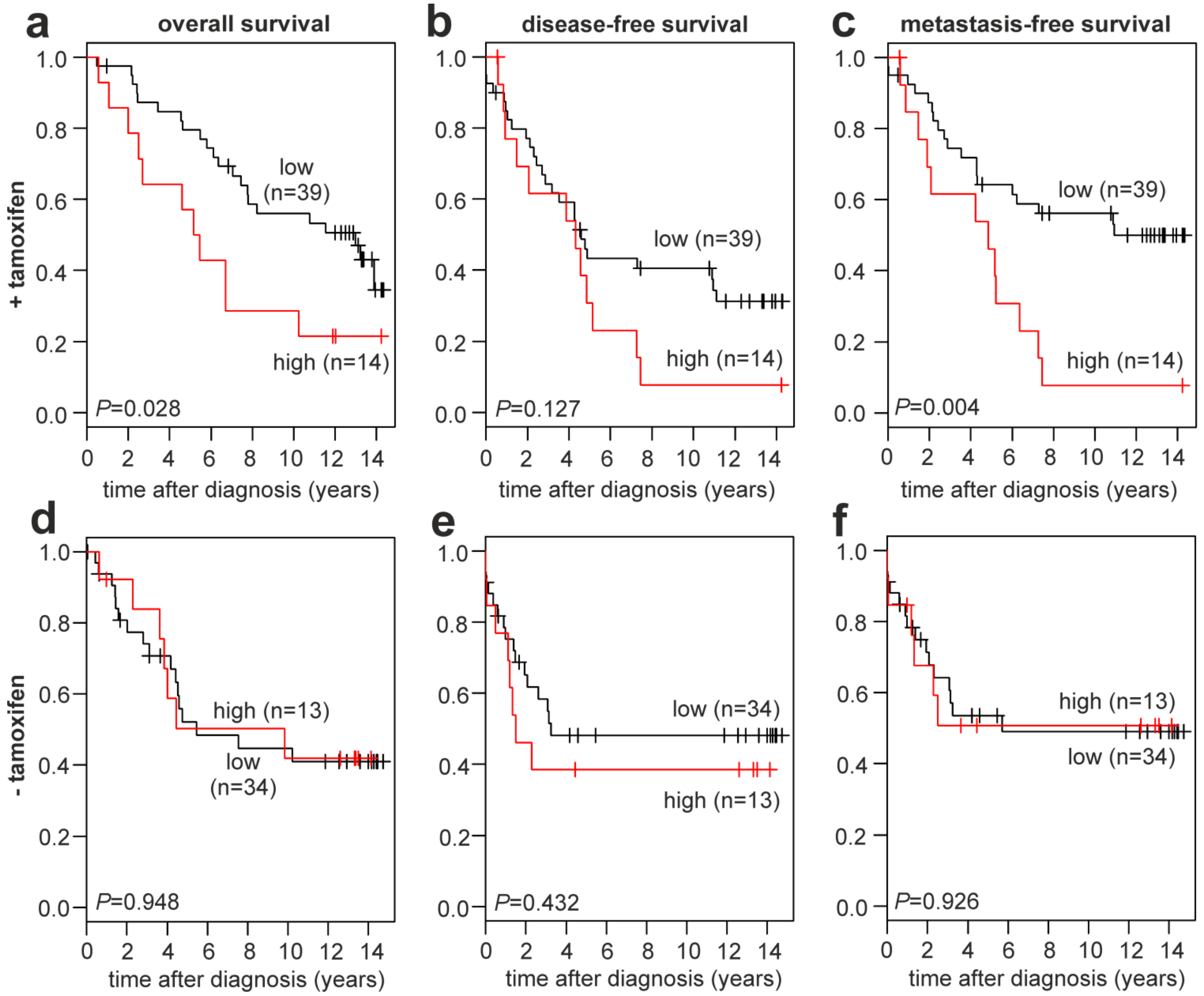

Supplementary Fig. 6 Association of *CYP19A1* mRNA expression with the survival of human breast cancer patients. Kaplan-Meier analyses of the overall survival (a, d), disease-free survival (b, e) and metastasis-free survival (c, f) in patients treated with tamoxifen (a-c; n=53) and patients not treated with tamoxifen (d-f; n=47) are shown. Patient subgroups with high and low *CYP19A1* expression and their numbers (n) are indicated in each panel.
